# Mesothelin promotes epithelial-to-mesenchymal transition and tumorigenicity of human lung cancer and mesothelioma cells

**DOI:** 10.1186/s12943-017-0633-8

**Published:** 2017-03-14

**Authors:** Xiaoqing He, Liying Wang, Heimo Riedel, Kai Wang, Yong Yang, Cerasela Zoica Dinu, Yon Rojanasakul

**Affiliations:** 1 Department of Pharmaceutical Sciences, West Virginia University, 1 Medical Center Drive, Morgantown, WV 26506 USA; 2 HELD, National Institute for Occupational Safety and Health, CDC, 1095 Willowdale Road, Morgantown, WV 26505 USA; 3 Department of Biochemistry, West Virginia University, Morgantown, USA; 4 Department of Chemical and Biochemical Engineering, West Virginia University, 395 Evansdale Drive, Morgantown, WV 26506 USA; 5 WVU Cancer Institute, 1 Medical Center Drive, Morgantown, WV 26506 USA

**Keywords:** Mesothelin, Lung cancer, Mesothelioma, Epithelial to mesenchymal transition, Cancer stem cells, Circulating tumor cells, Metastasis

## Abstract

**Background:**

Lung cancer and pleural mesothelioma are two of the most deadly forms of cancer. The prognosis of lung cancer and mesothelioma is extremely poor due to limited treatment modalities and lack of understanding of the disease mechanisms. We have identified mesothelin as a potentially unique therapeutic target that as a specific advantage appears nonessential in most cell types. Mesothelin (MSLN), a plasma membrane differentiation antigen, is expressed at a high level in many human solid tumors, including 70% of lung cancer and nearly all mesotheliomas. However, the role of MSLN in the disease process and underlying mechanisms is largely unknown.

**Methods:**

ShRNA knockdown and overexpression of MSLN were performed in human cancer cell lines and corresponding normal cells, respectively. Tumorigenic and metastatic effects of MSLN were examined by tumor sphere formation, migration, and invasion assays in vitro, as well as xenograft tumor assay in vivo. EMT and CSCs were detected by qPCR array, immunoblotting and flow cytometry.

**Results:**

MSLN plays a key role in controlling epithelial-to-mesenchymal transition (EMT) and stem properties of human lung cancer and mesothelioma cells that control their tumorigenicity and metastatic potential. Firstly, MSLN was found to be highly upregulated in non-small cell lung cancer (NSCLC) patient tissues and in lung carcinoma and mesothelioma cell lines. Secondly, genetic knockdown of MSLN significantly reduced anchorage-independent cell growth, tumor sphere formation, cell adhesion, migration and invasion in vitro, as well as tumor formation and metastasis in vivo. Thirdly, ectopic overexpression of MSLN induced the malignant phenotype of non-cancerous cells, supporting its role as an oncogene. Finally, mechanistic studies revealed that knockdown of MSLN reversed EMT and attenuated stem cell properties, in addition to inhibiting tumor growth and metastasis.

**Conclusions:**

These results indicate an essential role of MSLN in controlling EMT and stem cell properties of human lung cancer and mesothelioma cells. Since EMT is an important process in tumor progression and metastasis, and MSLN is nonessential in most normal tissue, our findings on MSLN may provide new insights into the disease mechanisms and may aid in the development of novel targeted therapy for lung cancer and mesothelioma.

## Background

Lung cancer is the leading cause of cancer death in the United States for both men and women. Estimated new cases and deaths for 2016 are projected to 224,390 and 158,080, respectively, and account for nearly 27% of all deaths from cancer [1]. Malignant mesothelioma is one of the most aggressive forms of cancer with an average survival period of less than one year after diagnosis [2]. Although smoking rates (a risk factor for lung cancer) have decreased over the years and asbestos (a major cause of mesothelioma) usage in construction has been prohibited, the incidence of lung cancer and mesothelioma is still high, possibly due to their long latency period of development after initial exposure and the complexity and diversity of new carcinogens [3, 4]. Despite significant advances in treatment management, the prognosis of lung cancer and mesothelioma remains very poor due to limited treatment options and lack of understanding of the disease mechanisms. Thus, identifying the key underlying molecular mechanisms of oncogenesis is essential for early detection and treatment of the diseases.

Mesothelin (MSLN) is a membrane-bound protein with unclear functions. The *Mesothelin* gene encodes a 69-kDa precursor protein that is cleaved into a 31-kDa secreted fragment called megakaryocyte potentiating factor (MPF), and a 40-kDa membrane-bound protein termed mesothelin (MSLN), which is a glycoprotein anchored to the plasma membrane by a glycophosphatidyl inositol (GPI) domain [5, 6]. MSLN is physically undetectable in most normal tissues except mesothelial cells of the peritoneal and pleural cavities and pericardium. However, MSLN is expressed at a high level in almost all mesothelioma and many solid tumors such as in lung cancer (60–70%), pancreatic cancer (80–85%), cholangiocarcinoma (60–65%), ovarian cancer (60–65%), gastric cancer (50–55%), colon cancer (40–45%), breast cancer (25–30%), and endometrial cancer (20–25%) [7]. Because of its prevalence in cancers, MSLN has recently been targeted for immunotherapy [7], while the soluble MSLN fragment has been investigated as a biomarker for cancer diagnosis [8]. Despite extensive studies of MSLN as a potential diagnostic and therapeutic target, neither the physiologic role of MSLN nor its pathological mechanism in cancer is well defined. In lung cancer, accumulating evidence indicates that high expression of MSLN is correlated with poor patient’s overall prognosis and relapse-free survival [9]. Preclinical studies showed that MSLN is involved in cell proliferation, anoikis resistant and survival [10–12], and its downregulation promotes drug-induced apoptosis and chemosensitivity [13, 14].

Epithelial to mesenchymal transition (EMT) results in physiological and phenotypic changes where epithelial cells acquire a mesenchymal phenotype. They break down cell-cell and cell-extracellular matrix connections that facilitate their translocation through the extracellular matrix to reach areas of new organ formation. Cancer cells adopt EMT process in the conversion of early stage tumors into dedifferentiated and more malignant states [15]. EMT plays a crucial role not only in tumor metastasis but also in tumor recurrence [16–18]. The role of MSLN in tumor formation and metastasis of lung cancer and mesothelioma or any role in EMT and cancer stem cell (CSC) regulation is largely unknown.

In this study, we investigated the role of MSLN in lung cancer and mesothelioma by evaluating the effects of MSLN knockdown and overexpression on tumor growth and metastasis in a mouse model. We also assessed the consequences of genetically altered MSLN levels on EMT, the malignant phenotype, and stem properties of human lung carcinoma and mesothelioma cells. Our results demonstrate the essential role of MSLN in promoting EMT and stemness, as well as tumor formation and metastasis.

## Methods

### Patient tumor samples

Human lung tumor tissues were obtained from the Lung Cancer Biospecimen Resource Network (Charlottesville, VA, USA). Four adenocarcinoma and six squamous cell carcinoma specimens with correlated adjacent healthy tissues were prepared and tested as pairs.

### Cell lines and culture conditions

Non-tumorigenic human bronchial epithelial BEAS-2B cells were cultured in bronchial epithelial basal medium along with additives from Lonza Corporation (Walkersville, MD, USA). Human lung carcinoma alveolar epithelial A549 cells were cultured in Dulbecco’s modified Eagle medium (DMEM) supplemented with 5% fetal bovine serum (FBS), 100 units/ml penicillin and 100 μg/ml streptomycin (Gibco, Gaithersburg, MA, USA). Non-small cell lung cancer H460 cells were cultured in RPMI 1640 medium supplemented with 5% FBS and 100 units/ml penicillin/streptomycin. Human pleural mesothelial MeT5A cells were maintained in M199 medium (Life Technologies, Grand Island, NY, USA) with 5% FBS, 2 mM L-glutamine, 100 units/ml penicillin/streptomycin, 1 μg/ml EGF, and 50 μg/ml hydrocortisone. Human pleural mesothelioma H2052 cells were cultured in RPMI 1640 medium supplemented with 10% FBS, and 100 units/ml penicillin/streptomycin. All cells used in this study were obtained from ATCC (Manassas, VA, USA) and were cultured in a humidified atmosphere of 5% CO_2_ at 37 °C.

### Generation of stable MSLN knockdown cell lines

Stable MSLN knockdown lines of H460 and H2052 cells, and their respective vector control lines, were generated via shRNA lentiviral vectors with four shMSLN or scrambled shRNA (OriGene Technologies, Rockville, MD, USA), according to the manufacturer’s instructions. Transfected cells were selected with 1–5 μg/ml of puromycin. Single clones of shMSLN and shRNA controls were verified by Western blotting.

### Overexpression of MSLN

MSLN expression plasmid, pAdEasy-MSLN-iCre-HA-Flag (plasmid#31305), was obtained from Addgene (Cambridge, MA, USA). The plasmid was amplified with a DNA midi kit (Qiagen, Hilden, Germany). 2 μg of MSLN plasmid DNA were transiently transfected into Met5A cells with FuGene HD transfection reagent (Promega, Madison, WI, USA). Functional assays were performed 24 h after the transfection.

### Cell proliferation

MSLN knockdown and shRNA control cells were seeded at a density of 1.5 × 10^4^ cells per well in 100 μl media in a 96-well plate (Fisher, Waltham, MA, USA). After 24, 48, and 72 h, 20 μl of CellTiter 96 Aqueous One Solution (Promega, Madison, WI, USA) were added to each well and the cells were incubated at 37 °C for an additional 3 h. Viable cells cleaved the reagent’s tetrazolium salt to a soluble formazan dye, resulting in a color change proportional to the number of live cells. Absorbance was measured at 490 nm with a reference wavelength at 630 nm using a BioTek plate reader (BioTek, Winooski, VT, USA).

### Cell surface area measurements

Cells were stained with CellTracker™ Green CMFDA dye or CellTracker™ Red CMTPX dye (Thermo Fisher Scientific, Pittsburgh, PA, USA) and seeded into glass chambers at the density of 1 × 10^5^/ml. After culturing for 24 h, the cells were fixed with 4% paraformaldehyde and imaged by a Nikon Ti Eclipse fluorescence microscope. The surface area of cells was measured using Image J software (http://imagej.nih.gov/ij/). A minimum of 200 cells were analyzed for each group.

### Soft agar colony formation assay

Control shRNA and shMSLN knockdown cells (2,500 cells) were suspended in 0.5 ml culture medium and mixed with an equal amount of 0.7% agar to a final agar concentration of 0.35%. The mixed cell-agar suspensions were immediately plated onto 6-well plates coated with 0.5% agar in culture medium. Colonies were examined under a light microscope after 2 weeks of culture.

### Tumor sphere formation assay

Tumor sphere formation assay was performed under non-adherent and serum-free conditions. Briefly, 5,000 cells were suspended in 0.8% methylcellulose-based serum-free medium (Stem Cell Technologies, Vancouver, Canada) supplemented with 20 ng/ml epidermal growth factor (BD Biosciences, San Jose, CA, USA), 10 ng/ml basic fibroblast growth factor and 5 μg/ml insulin (Sigma-Aldrich, St Louis, MO, USA) in ultra-low adherent 6-well plates (Corning Incorporated, Kennebunk, ME, USA). Cells were cultured for two weeks after which tumor spheres were examined under a light microscope. In order to assess self-renewal property of the cells, spheres were collected by gentle centrifugation, dissociated into single cell suspensions, filtered and cultured under the same conditions to form secondary spheres.

### Cell migration and invasion assays

Cell migration was determined by using a 24-well Transwell® unit (Thermo Fisher Scientific, Pittsburgh, PA, USA) with a polyvinylidene difluoride filter (8-μm pore size). Cell invasion was assayed by using a BD Matrigel® invasion chamber (BD Biosciences, Franklin Lakes. NJ, USA). Briefly, 1.5 × 10^**4**^ cells per well (migration) or 3 × 10^**4**^ cells per well (invasion) were seeded into the upper chamber of the Transwell® unit in serum-free medium. The lower chamber was filled with a normal growth medium containing 5% FBS. Chambers were incubated at 37 °C in a 5% CO_2_ atm for 48 h. Non-migrating or non-invading cells in the inside of the Transwell® inserts were removed with a cotton swab. Cells that migrated or invaded to the underside of the membrane inserts were fixed and stained with Diff-Quik (Dade Behring, Newark, DE, USA). Inserts were visualized and scored under a light microscope (Leica DM, Deerfield, IL, USA). The number of migrating and invading cells from ten random fields were scored.

### Pathway specific PCR array

Total RNA from control and MSLN knockdown cells were isolated using a Qiagen RNA mini kit (Qiagen, Valencia, CA, USA) and reverse-transcribed into single stranded cDNA. Differential expression of EMT genes was analyzed using a RT^2^ profiler PCR array: EMT Pathway (Qiagen, Valencia, CA, USA) following the manufacturer’s instructions. Data analysis was performed online at www.SABiosciences.com/pcrarraydataanalysis.php.

### Immunoblotting

Cells were washed with PBS and lysed on ice with modified RIPA buffer containing protease and phosphatase inhibitor mixtures (Roche Molecular Biochemicals, Indianapolis, IN, USA) for 30 min. The lysates were briefly sonicated and centrifuged at 14,000 × g for 20 min. Cell lysates (40 μg protein) were fractionated by 10% sodium dodecyl sulfate-polyacrylamide gel electrophoresis (SDS-PAGE) and transferred onto polyvinylidene difluoride membranes (Bio-Rad Laboratories, Hercules, CA, USA). The transfer membranes were blocked for 1 h in 5% nonfat dry milk in TBST (25 mM Tris–HCl, pH 7.4, 125 mM NaCl, 0.05% Tween 20) followed by treatment with primary antibodies at 4 °C overnight with gentle shaking. Membranes were washed three times with TBST for 10 min each, followed by incubation with horseradish peroxidase-conjugated secondary antibodies for 1 h at room temperature. Protein bands were visualized using enhanced chemiluminescence detection reagents from Millipore (Millipore Corporation, Billerica, MA, USA). Actin was used as a loading control and the data were quantified using Image J densitometry software.

### Immunohistochemistry staining

Lung and liver tissue sections in paraffin were deparaffinized and rehydrated. Antigens were retrieved with 10 mM sodium citrate solution in a microwave for 20 min. The slides were then blocked with 3% BSA/0.1% Tween in 1 × PBS blocking buffer for 1 h, and were incubated with anti-human MSLN antibody (Abcam, Cambridge, MA, USA) (1:500) or anti-human mitochondria antibody (EMD Millipore, Temecula, CA, USA) (1:100) overnight at 4 °C. After washing with PBS three times, the slides were incubated with biotinylated secondary antibodies for another hour, followed by ABC reagent (Vector Laboratories, Burlingame, CA, USA) and detected with a DAB kit (Vector Laboratories, Burlingame, CA, USA). After color development, the slides were counterstained with hematoxylin, dehydrated, and mounted with Permount mounting solution. Images were taken using a light microscope with Olympus cellSens Dimension software.

### Flow cytometric ALDH activity assay

The Aldefluor^TM^ kit (StemCell Technologies, Durham, NC, USA) was used to analyze and isolate the cell population with high ALDH enzymatic activity. Cells were suspended in Aldefluor assay buffer containing ALDH substrate (BODIPY-aminoacetaldehyde, 1 mmol/l per 1x10^6^ cells) and incubated for 40 min at 37 °C. As a control, an aliquot of the sample was treated with 50 mmol/l of the specific ALDH inhibitor diethylaminobenzaldehyde (DEAB).

### Tumor xenograft mouse model

Animal care and experimental procedures described in this study were performed in accordance with the Guidelines for Animal Experiments at West Virginia University with the approval of the Institutional Animal Care and Use Committee (IACUC #15-0702). Immunodeficient NOD/SCID gamma mice, strain NOD.Cg-Prkdcscid Il2rgtm1Wjl/SzJ (NSG; Jackson Laboratory, Bar Harbor, ME, USA), were maintained under pathogen-free conditions within the institutional animal facility. Food and water were given ad libitum. Mice (6/group) were subcutaneously injected with 1 × 10^6^ cells of H460 or H2052 with shMSLN stable knockdown or shRNA control cells suspended in 100 μl of ExtraCel® hydrogel (Advanced BioMatrix, San Diego, CA, USA). Mice were inspected daily for any signs of distress such as weight loss, hunching, failure to groom, and red discharge from the eyes. At the end of the experiments, mice were euthanized and tumors were dissected and weighted. Metastatic nodules were counted from the surface of the intestine, liver, and lungs. Tumor specimens were cut into 5-μm sections and stained with hematoxylin and eosin (H&E) to confirm cancer morphology and metastasis in the organs. All tissue sectioning and staining procedures were performed at the West Virginia University Pathology Laboratory for Translational Medicine.

### Statistical analysis

Results are expressed as means ± s.d. from three or more independent experiments to ensure adequate power (>80%). Differences between groups were assessed by one-way analysis of variance (ANOVA) followed by Student’s *t* test. For all analyses, two-sided P values of ≤ 0.05 were considered statistically significant.

## Results

### MSLN expression in human lung tumor samples and cell lines

**Fig. 1:**
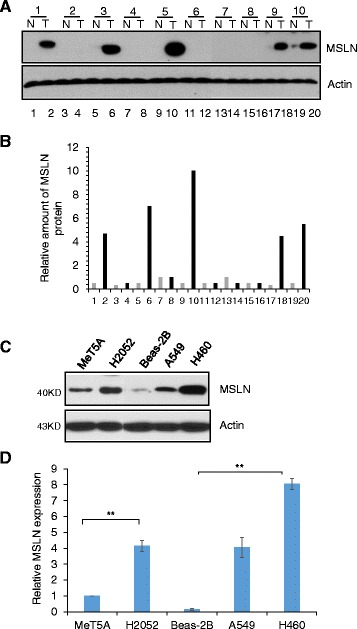
MSLN is upregulated in lung cancer patients and cell lines. **a** Western blot for MSLN in pairs of lung tumor tissue (T) and normal tissue control (N) from the same patient. Pairs 1–6 are large cell carcinomas, pairs 7–9 are squamous cell carcinomas, and pair 10 is adenocarcinoma. **b** Quantification of tumor MSLN expression, relative to actin. **c** Western blot and **d** quantification of MSLN in normal mesothelial (MeT 5A), malignant mesothelioma (H2052), normal epithelial (BEAS-2B), and cancerous epithelial (A549 and H460) cells

To study the role of MSLN in lung cancer and mesothelioma, we first evaluated the expression level of MSLN in human cancer patients and cell lines. Ten pairs of human lung cancer samples with adjacent normal tissue controls were examined by Western blot analysis. MSLN protein levels were not detectable in any of the normal tissues, but clearly in 5 out of 10 lung tumor tissues, i.e. sample pair #1, 3, 5, 9, and 10 in Fig. 1a and b (#1 large cell carcinoma, stage III, #2 large cell carcinoma, stage III, #3 large cell carcinoma, stage III, #4 large cell carcinoma, stage II, #5 large cell carcinoma, stage IV, #6 large cell carcinoma, stage III, #7 squamous cell carcinoma, stage III, #8 squamous cell carcinoma, stage III, #9 squamous cell carcinoma, stage III, #10 adenocarcinoma, stage II). The increase in MSLN expression in the tested tissue samples ranged from 2–10 fold over matched controls. We also tested the expression of MSLN in established human lung cancer cell lines (H460 and A549) and mesothelioma cell line (H2052). When compared to normal (non-cancer) human lung epithelial cell line (BEAS-2B) and mesothelial cell line (MeT 5A), expression of MSLN was highly elevated in the cancer cell lines (Fig. 1c and d), suggesting a carcinogenic role of MSLN in lung cancer and mesothelioma.

### MSLN knockdown inhibits the cancer phenotype of human lung epithelial and mesothelial cells

**Fig. 2:**
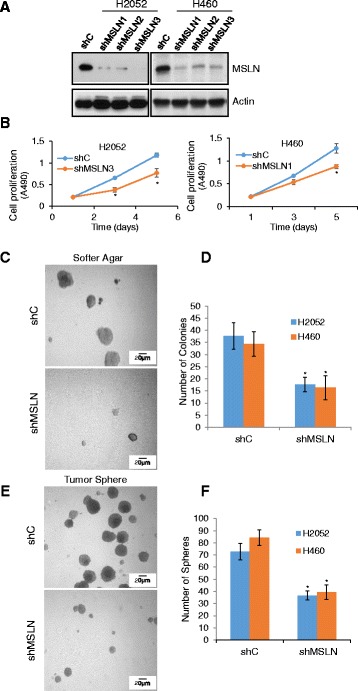
MSLN knockdown reduces soft agar colony formation and tumor sphere formation. **a** Western blot of MSLN in several stable knockdown clones by shMSLN RNA. **b** Proliferation of control (shC) and MSLN knockdown (shMSLN) H2052 and H460 cells, means ± s.d. (*n* = 6), *P < 0.05. **c** Representative images and **d** quantification of soft agar colony formation of shC and shMSLN H2052 and H460 cells. **e** Representative images and **f** quantification of tumor sphere formation of shC and shMSLN H2052 and H460 cells. *P < 0.05 vs shC, *n* = 6

To determine the functional role of MSLN in lung cancer and mesothelioma, two MSLN knockdown lung carcinoma H460 and mesothelioma H2052 cell lines were generated by stably transfecting the cells with short-hairpin (sh) RNA against MSLN (shMSLN). A scrambled shRNA was used to generate vector-transfected control (shC) cell lines. Several individual, stable clones of the transfected cells were selected and analyzed for MSLN expression by Western blotting. Representative knockdown clones are presented in Fig. 2a. These clones were analyzed for cell growth, colony formation, and tumor sphere formation. Figure 2b shows that the MSLN knockdown (shMSLN) clones exhibited a slower rate of proliferation than their vector (shC) controls in both H460 and H2052 cell lines. Soft agar colony formation assay indicated that the shMSLN cells formed substantially smaller and fewer colonies than the control shC cells (Fig. 2c and d). Likewise, shMSLN cells formed smaller and fewer of tumor spheres than control cells (Fig. 2e and f). These results strongly support the pro-carcinogenic role of MSLN in the tested cell system. Since tumor sphere formation commonly serves as one of the indicators of cancer stem-like cell (CSC) formation, the results of this study also suggest the role of MSLN in CSC regulation, which is supported by our subsequent studies.

**Fig. 3:**
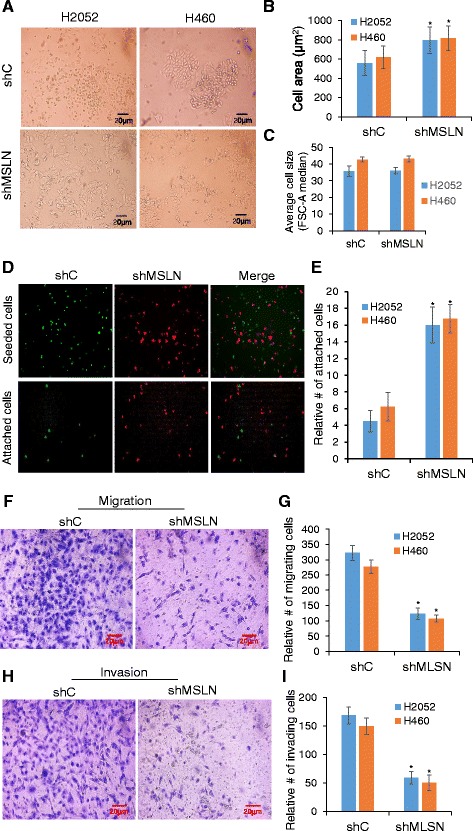
MSLN knockdown reduces mesenchymal like morphology, cell-substrate adhesion, cell migration and invasion*.* **a** Representative images of cell morphology of shC and shMSLN clones. **b** Digital quantification of the cell surface area using image J software, means ± s.d., *P < 0.05 vs shC. **c** Average size of shC and shMSLN cells from flow cytometry FSC-A median. **d** Representative images of fluorescently labeled cells before and after washing to remove non-adherent cells. *Green* fluorescent shC cells and *red* fluorescent shMSLN cells. **e** Quantification of the relative number of attached cells after washing normalized by the number before washing, *P < 0.05 vs shC. **f** Representative images and **g** quantitation of migration of shC and shMSLN cells from Transwell, stained with Diff-Quik. **h** Representative images and **i** quantitation of invasion of shC and shMSLN cells from Matrigel-coated Transwell, stained with Diff-Quik. *P < 0.05 vs shC

Morphologically, the shMSLN cells exhibited epithelial morphology and appeared more flat and more adherent to the culture plate than the shC cells, which displayed a spindle-like shape and were less flat in appearance similar to their parental lines, (Fig. 3a). Quantitative analysis of cell surface area by CellTracker™ Green CMFDA dye or CellTracker™ Red CMTPX dye labeling and digital imaging indicated that the shMSLN cells displayed about 20% more surface area than the shC cells when attached (Fig. 3b). In order to rule out the possibility that MSLN knockdown increases cell size thus affecting cell surface area upon adherence to culture surface, a comparison of cell size was performed using flow cytometry. As shown in Fig. 3c, there was no statistical difference in the FSC-A median between shC and shMSLN groups. To test if the shMSLN cells might adhere more tightly to the substrate than control cells, we analyzed their adhesion property using CellTracker™ fluorescent probes. Green CMFDA-labeled shC cells and red CMTPX-labeled shMSLN cells were mixed at equal numbers and seeded onto a 24-well culture plate. After a 3-h incubation period, unattached cells were rinsed out and attached cells were visualized and quantified by fluorescence microscopy. As depicted in Fig. 3d and e, the green shC and red shMSLN cells were comparable in number at the beginning of the experiments. However, after rinsing the red shMSLN cells outnumbered the green shC cells by approximately 2–3 fold, indicating that they were more tightly bound to the substrate than the control cells. This result implies that MSLN knockdown cells are more epithelial-like and likely to localize in the primary tumor as compared to control cells which are more mobile and likely to metastasize to other tissues. Experiments to assess the motility of MSLN knockdown cells using Transwell**®** assays showed that the shMSLN cells were indeed less migratory and less invasive through extracellular matrix than the control shC cells (Fig. 3f-i).

**Fig. 4:**
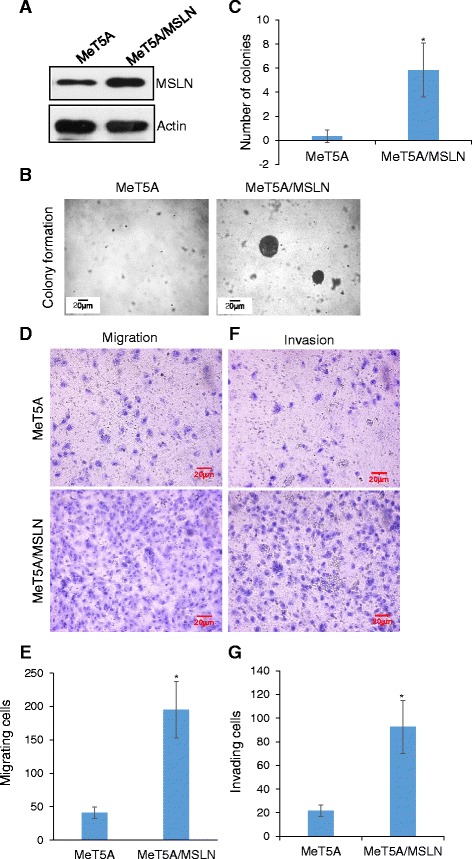
Overexpression of MSLN promotes colony formation, cell migration, and invasion. **a** Western blot analysis of MSLN overexpression in mesothelial MeT5A cells (MeT5A/MSLN). **b** Representative images and **c** quantification of soft agar colony formation of MeT5A and MeT5A/MSLN cells. *P < 0.05, *n* = 4. **d** Representative images and **e** quantification of migration of MeT5A and MeT5A/MSLN cells, *P < 0.05, *n* = 4. **f** Representative images and **g** quantification of invasion of MeT5A and MeT5A/MSLN cells from Matrigel-coated Transwell, *P < 0.05, *n* = 3

To support the above findings, we performed gene overexpression experiments comparing the effects of MSLN overexpression on anchorage-independent growth, migration, and invasion in non-cancerous MeT5A mesothelial cells. The results showed that the control MeT5A cells formed no or very few small colonies in soft agar, whereas the MSLN overexpressing cells (MeT5A/MSLN) formed multiple large colonies (Fig. 4,a-c). Cell motility studies also showed that the MSLN overexpressing cells were more migratory and invasive than the control cells (Fig. 4d-g). Together, these findings support the tumorigenic/metastatic role of MSLN in the tested cell systems.

### MSLN promotes tumorigenesis and metastasis in vivo

**Fig. 5:**
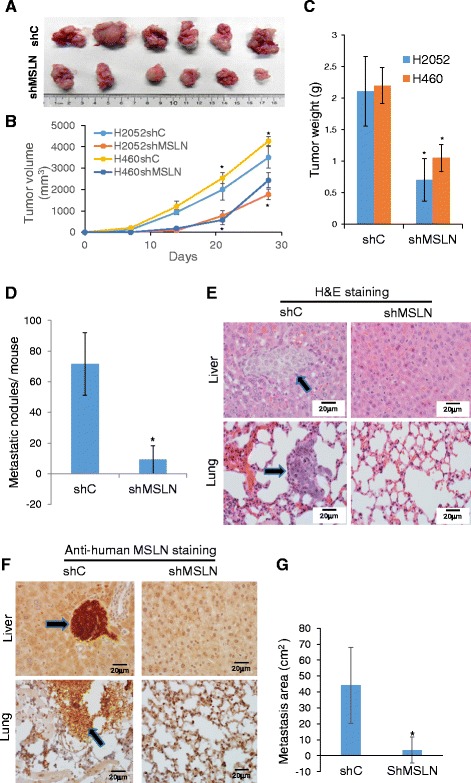
MSLN knockdown reduces tumor formation and metastasis*.* NSG mice were injected subcutaneously with 1 × 10^6^ cells of shMSLN or shC H2052 and H460 cells and analyzed for tumor formation and metastasis after 4 weeks. **a** Image of tumors from injection site of shC and shMSLN groups. **b** Tumor growth curves of local site tumor volume, means ± s.d. (*n* = 6), *P < 0.05 (**c**) Quantitation of local site tumor weights, means ± s.d. (*n* = 6), *P < 0.05. **d** Number of thoracic and abdominal surface metastatic nodules, means ± s.d. (*n* = 6), *P < 0.05. **e** H&E staining of pulmonary and hepatic tissue sections, arrows denote metastatic tumor nodules. **f** Immunohistochemistry staining of pulmonary and hepatic tissue sections with anti-human MSLN antibody, *arrows* denote human MSLN positive nodules. **g** Quantification of metastatic tumor areas, *P < 0.05

To verify the in vitro observations, we conducted in vivo experiments assessing the effects of MSLN knockdown on tumor formation and metastasis using a xenograft mouse model. MSLN knockdown (shMSLN) and control (shC) mesothelioma cells were injected into NSG mice subcutaneously, and tumor formation and metastasis were determined. Figure 5a, b, and c) shows that while the control shC mice developed faster and larger tumors, the shMSLN mice formed substantially smaller tumors. The shMSLN mice also formed fewer numbers of metastatic nodules at distant sites, which were abundant in shC mice (Fig. 5d). The metastatic nodules were observed in the liver and lungs of all mice in the shC group, whereas only one in six mice in the shMSLN group developed such nodules. Histopathological analysis of liver and lung tissue sections from the shC and shMSLN mice confirmed this finding (Fig. 5e). Immunohistochemistry staining of the liver and lung tissue sections with anti-human MSLN antibody confirmed that the tumor nodules were of human origin (Fig. 5f). Tumor area analysis of the tissue sections revealed a substantial reduction in liver and lung tumors in the shMSLN group compared to the shC group (Fig. 5g).

### MLSN regulates the expression of multiple EMT genes and controls cancer stem cell trait

**Fig. 6:**
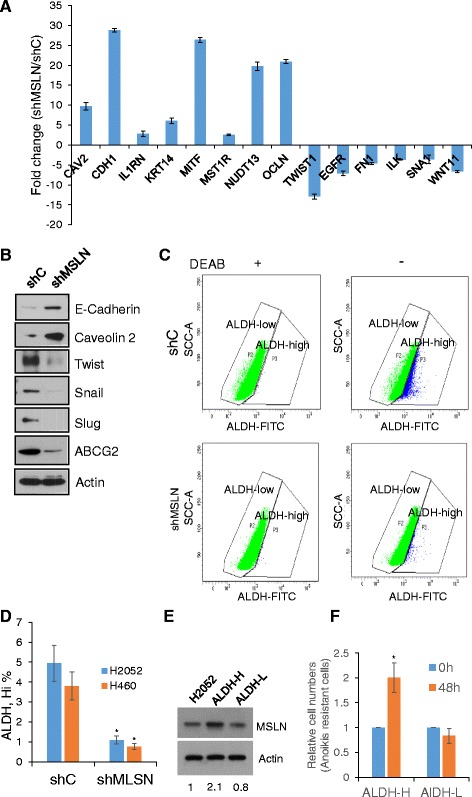
MSLN knockdown reduces EMT and cancer stem cell traits. **a** Relative fold change of gene expression from an EMT pathway specific PCR array. Data represent candidate transcripts exceeding two-fold *up* or *down* regulation in shC and shMSLN cells. **b** Western blot analysis of representative EMT and CSC markers. Actin was used a loading control. **c** Flow cytometry graphical representation of ALDH activity in shC and shMSLN cells with or without ALDH inhibitor DEAB. **d** Quantification of ALDH activity, means ± s.d. (*n* = 3), *P < 0.05. **e** Western blot analysis of MSLN expression in ALDH activity high (ALDG-H) and ALDH activity low (ALDH-L) fractions after flow cytometric ALDH sorting. **f** ALDH-H fraction has a relative higher number of anoikis resistant cells. Sorted ALDH-H and ALDH-L cells were seeded in low attachment plates and cell numbers were counted at 0 and 48 h, means ± s.d. (*n* = 3), *P < 0.05

It has been proposed that cancer cells undergo EMT during tumor progression and metastasis, in which they lose epithelial characteristics and acquire invasive properties and stem features [19, 20]. Cancer stem cells are thought to be generated from EMT as a key driver of tumor growth, metastasis, and relapse. To investigate the mechanisms of MSLN-driven tumorigenesis and metastasis, an EMT pathway specific PCR array was employed to identify potential driver genes. Eighty four key genes known to be important in EMT regulation were investigated in shMSLN and shC cells (Fig. 6a). Knockdown of MSLN strongly affected the expression of several EMT pathway specific genes, including 8 epithelial differentiation-related genes which were upregulated, and 6 growth factor related genes which were downregulated, all at least 2 fold when compared to shC. CDH1, which encodes epithelial cadherin (E-cadherin), an epithelial specific protein controlling cell-cell adhesion [21], was upregulated by 28.5 fold, whereas CAV2 which encodes caveolin-2, a potent tumor suppressor controlling lipid metabolism, growth, and apoptosis [22], was upregulated by 9 fold in shMSLN cells. Likewise, MITF, which encodes microphthalmia-associated transcription factor, also known as class E basic helix-loop-helix protein 32 (bHLHe32), was upregulated by 26 fold. MITF has been reported to be an important factor in controlling mesenchymal to epithelial transition [23]. Other genes that were upregulated include NUDT13 or nudix-type hydrolase 13, and OCLN which controls epithelial tight junctions [24]. On the other hand, a number of oncogenes and growth factors were downregulated in shMSLN cells. For example, TWIST, a key transcription factor regulating EMT [24], was downregulated 13 fold, as other factors including EGF, FN1 and snail1, which control tumor growth and stemness, and were reduced by 3–7 fold. The pathway specific PCR results were verified by Western blotting, which confirmed that MSLN knockdown upregulated the epithelial markers (E-cadherin and caveolin-2) and downregulated stem cell/EMT markers (twist, snail, slug, and ABCG2) (Fig. 6b).

Aldehyde dehydrogenase (ALDH) has frequently been used as a CSC marker, and its high activity has been associated with self-renewal and drug resistant phenotypes of cancer cells [25, 26]. Flow cytometric analysis of ALDH activity by Aldefluor**®** assay revealed that knockdown of MSLN significantly reduced the ALDH activity of lung carcinoma (H460) and mesothelioma (H2052) cells (Fig. 6, c and d). Furthermore, analysis of ALDH activity-high and ALDH activity-low cells after flow cytometry sorting showed that the ALDH high cells expressed a higher level of MSLN and were more resistant to anoikis (detachment-induced cell death) as compared to the ALDH low cells (Fig. 6e and f). These results suggest that the regulation of EMT and CSC related genes may underlie the pro carcinogenic mechanism of MSLN.

## Discussion

Although MSLN appears to be non-essential in normal tissues since MSLN knockout mice exhibit no detectable malfunction in tissue development, reproduction, and blood cell count [27], clinical studies have shown that high MSLN expression correlates with tumor aggressiveness in many solid tumors [7]. Consistent with previous studies, our presented results indicate an upregulation of MSLN in human lung tumor tissues and in lung carcinoma and mesothelioma cell lines. Knockdown of MSLN in these cell lines reversed their malignant phenotype as indicated by soft agar colony formation, tumor sphere formation, and cell migration and invasion assays (Figs. 2 and 3) as well as tumor formation in animals (Fig. 5). In non-cancerous cells, overexpression of MSLN promoted a malignant phenotype as indicated by anchorage-independent growth and cell migration and invasion assays (Fig. 4). Together, these results strongly support the general pro-carcinogenic role of MSLN, which is supported by clinical observations that show a linkage between MSLN expression and tumorigenesis in lung, breast, and pancreatic cancers [9, 19, 28, 29].

Metastasis is the primary cause of death in patients with advanced cancer. In addition to regulating tumor formation, our data also suggest the role of MSLN in controlling metastasis. Supporting this notion knockdown of MSLN effectively inhibited liver and lung metastasis (Fig. 5) and migration and invasion of tumor cells (Fig. 3). Metastasis is a highly complex process, closely associated with EMT, the phenotypic transformation of well-differentiated epithelial carcinoma into a mesenchymal-like state. This transformation provides cancer cells with the ability to breakdown epithelial cell-cell tight junctions, invade extracellular matrix basement membranes, and enter the circulation to become circulating tumor cells (CTCs). Previous studies showed that CTCs possess both EMT and CSC characteristics [30, 31]. Cells from primary tumors were found to express a combination of mesenchymal and epithelial markers, whereas CTCs express predominantly mesenchymal markers [32]. Our study showed for the first time that MSLN regulates EMT, and possibly CTCs and CSCs, which may be responsible for tumorigenesis and metastasis. Lung cancer (H460) and mesothelioma (H2052) cells exhibit some degree of mesenchymal and CTC phenotypes in terms of morphology, basement membrane attachment, and migratory and invasive activities. Knockdown of MSLN dramatically changed the morphology of the cells from a mesenchymal spindle-like shape to epithelial-like shape, increased their adhesion and spreading on cell culture substrata, and decreased their migration and invasion (Fig. 3). These phenotypic changes decreased the likelihood of the cells to exit the tissue and become CTCs, and to metastasize to other tissues.

PCR array and western blot analyses were used to characterize EMT and CSC markers in control and MSLN knockdown cells. The control cells expressed a high level of mesenchymal and CSC markers, whereas the shMSLN cells expressed predominantly epithelial markers (E-cadherin, caveolin, and occludin) and a low level of mesenchymal and CSC markers (Twist, EGFR, Snail, Slug, ABCG2, and ALDH activity (Fig. 6). Recent studies have shown that EMT is a key driver of CSC formation which controls tumor progression and the treatment response [16, 32, 33]. The low adherent (trypsin sensitive) subpopulation of breast and colon cancer cells exhibited EMT and stem properties with increased ALDH activity [34]. A cell tracking method demonstrated a dynamic change from EMT, CTCs to CSCs and distant metastasis in vivo in pancreatic cancer [35]. In mesothelioma cells, we present that knockdown of MSLN reversed the EMT to MET phenotype and significantly reduced CSC markers and ALDH activity, which may contribute to the observed reduction in tumorigenicity and metastasis of the knockdown cells.

MSLN appears to regulate EMT through multiple pathways and downstream targets. For example, knockdown of MSLN promoted the epithelial phenotype by up-regulating E-cadherin, cytokeratins, claudins, occludin, IL1RN, MITF, MSTIR, and NUDT, and by downregulating transcription factors such as Twist, Snail1, as well as fibronectin, ILK, EGFR, and WNT11 (Fig. 6). E-cadherin, encoded by CHD1, is a calcium-dependent cell-cell adhesion glycoprotein. Loss of E-cadherin is associated with mesenchymal transition and metastatic activity of cancer cells [36]. Claudins and occludin are key components of tight junction proteins, which regulate epithelial/endothelial permeability [37] and directional migration [38]. Loss of occludin causes increased cell invasion, reduced adhesion, and impaired tight junction integrity in breast cancer tissues [39]. Cytokeratins are keratin-containing filaments that preserve cell structure and cell-cell adhesion. Twist is a key transcription factor involved in embryogenesis and development and regulates EMT and cell migration [40]. Snail belongs to a family of zinc-finger transcription factors that is essential for embryonic development and well-known to induce EMT [41]. The effect of MSLN on EMT may be cell-line dependent since a previous study by Wang et al. [11] showed that knockdown of MSLN in H2373 mesothelioma cell line did not affect E-cadherin expression but decreased β-catenin expression and increased Slug expression. Our morphological and functional assays confirmed that knockdown of MSLN in H2052 and H460 cells reversed their EMT phenotypes (Figs. 2 and 3), consistent with our EMT markers expression data (Fig. 6).

Induction of EMT is a highly complex process and involves several coordinated networks and signaling pathways. It is triggered by growth factors, such as transforming growth factor (TGF)-β, fibroblast growth factor (FGF), and epidermal growth factor (EGF). Binding of these growth factors to their respective surface receptors activates intracellular effector molecules and subsequently transcriptional activators such as snail and slug, which regulate functional molecules of EMT [42]. E-cadherin is a key target of Snail, Twist, and ZEB family members, and is often downregulated in aggressive carcinomas as a result of EMT induction [43]. Downregulation of E-cadherin weakens cell-cell adhesion, triggers cell migration from the primary tumor to systemic circulation, and promotes CSC formation and metastasis in distant organs [17, 44]. The impact of MSLN on several EMT and CSC regulatory genes that we have observed suggests that MSLN may act as a master regulator of EMT that controls both local invasion and distant metastasis.

## Conclusions

**Fig. 7:**
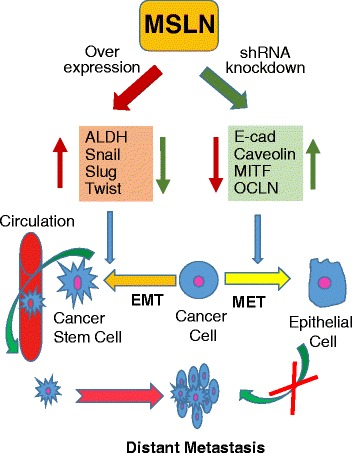
MSLN regulates EMT and cancer stem cell traits. MSLN knockdown upregulates epithelial and adhesion molecules, and downregulates mesenchymal and CSC regulatory genes that mitigate self-renewal, proliferation, dissemination, and metastasis of cancer cells. Overexpression of MSLN in normal cells stimulates anchorage-independent growth, migration and invasion

We provide new evidence for the role of MSLN in EMT regulation, tumorigenesis and metastasis. Knockdown of MSLN led to mesenchymal to epithelial transition and less aggressive behavior of lung carcinoma and mesothelioma cells. Such knockdown also resulted in a reduction of EMT and CSC markers and a parallel decrease in tumor growth and metastasis in animals. Our findings on MSLN, as schematically summarized in Fig. 7, could aid in the understanding of lung cancer progression and metastasis. Because of its importance in EMT and CSC regulation, MSLN could be a potential therapeutic target for advanced and recurrent lung cancer and mesothelioma. The finding that MSLN is nonessential in most tissues in mouse knockout studies supports its potential as unique therapeutic target.

## Abbreviations

CSCs: Cancer stem cells
CTCs: Circulating tumor cells
EMT: Epithelial-to-mesenchymal transition
MET: Mesenchymal-to-epithelial transition
MSLN: Mesothelin
